# Cryochemical Synthesis of Polymorphous Nanostructures of a Steroid Neurohormone

**DOI:** 10.3390/molecules22081378

**Published:** 2017-08-21

**Authors:** Yurii Morozov, Dmitry Chistyakov, Vladimir Chernyshev, Gleb Sergeev

**Affiliations:** 1 Department of Chemistry, M. V. Lomonosov Moscow State University, Moscow 119991, Russia; yunmor@mail.ru (Y.M.); vladimir@struct.chem.msu.ru (V.C.); gbs28@rambler.ru (G.S.); 2 Department of Fundamental Science, Bauman Moscow Technical State University, Moscow 105005, Russian; 3 Belozersky Institute of Physico-Chemical Biology, Moscow State University, Moscow 119991, Russia; 4 A. N. Frumkin Institute of Physical Chemistry and Electrochemistry RAS, Moscow 199071, Russian

**Keywords:** hormones, dehydroepiandrosterone (DHEA), new structures, polymorphism, nanoparticles, cryosynthesis

## Abstract

A new cryochemical strategy of producing nanoparticles and polymorphous nanostructures of drugs is used, which is based on the dynamic combination of high and low temperatures, gas and solid phases, and inert carrier gases. This technology is applied to the synthesis of nanoparticles of steroid neurohormone dehydroepiandrosterone (DHEA). We have optimized the conditions of synthesis of the new polymorphous DHEA structure, FVII. The molecules of DHEA in FVII structure are bound by hydrogen bonds via oxygen atoms. The grain size is 100 nm. It is shown that the yield and ratio of the resulting nanoforms of this hormone are determined by the nature and properties of the inert carrier gas. The highest yield and selectivity of FVII are observed when carbon dioxide is used as the carrier gas. In the case of helium, the FVII content decreases from 85 to 30% and other structures are formed. In experiments without carrier gas, nanoparticles are formed but no FVII is produced. The selectivity and the effect of carrier gas are considered on the basis of homogeneous and heterogeneous formation of nanoparticles and the relationship between particle selectivity and its activity. The synthesis of various polymorphous structures on the nanoscale is assumed to be the manifestation of the size effect in the synthesis of drugs.

## 1. Introduction

The therapeutic use of many modern drugs depends strongly on the methods of their introduction, their solubility, and bioavailability [1]. At present, these problems are often solved through the development of solid amorphous dispersions [2,3]. It is well known that the amorphous state is characterized by the high stored energy and, consequently, by the higher solubility and dissolution rate [3]. Attention is focused on systems that include, besides the drug itself, the additions of low-molecular compounds, e.g., amino acids [2]. The higher solubility and bioavailability of drugs in the form of crystals can also be associated with the formation of polymorphic modifications that can alter the physicochemical properties of the starting compounds [4]. The higher solubility and dissolution rate are traditionally associated with the decrease in the size of original substances [5]. Several versions of synthesis of drug nanoparticles were proposed [6]. The methods used most widely are those of dry and wet mechanical grinding [7,8]. Drawbacks of mechanical grinding are associated with high-energy consumption, wide size distribution of particles, and the presence of different compounds used for stabilizing the resulting dispersions and suspensions [9]. We have developed a novel strategy for the synthesis of drug nanoparticles, which is based on the dynamic combination of high and low temperatures, gas and solid phases, and inert carrier gases. The crystals of the original compounds are sublimated in the flow of heated inert carrier gas. The mixed flux of molecules is directed to the cold surface, where the temperature abruptly drops, the equilibrium shifts, which gives rise to strengthening of intermolecular interactions, induces homogeneous and heterogeneous nucleation, providing the growth of nanoparticles and the formation of stabilized structures. The proposed strategy requires no solvents and also allows scaling [10,11]. The cryochemical technology was used in the synthesis of nanoparticles of androstenediol [12], phenazepam [13], and the steroid neurohormone dehydroepiandrosterone (DHEA) [14]. The size of synthesized crystalline particles fit the interval from 40 to 200 nm. Besides producing nanoparticles, the strategy proposed made it possible to synthesize new polymorphous nanostructures of phenazepam and DHEA. The crystallographic parameters of these new DHEA structures can be found in [14]. In contrast to the known DHEA modifications, the new polymorphous modification DHEA VIII has a spiral chain structure in which DHEA molecules form hydrogen bonds only via oxygen atoms. The discovery of polymorphs in the form of nanostructures may open up new possibilities in the therapeutic use of traditional drugs [4]. The properties of polymorphous nanostructures of individual pharmaceutical substances determined by their synthetic conditions are virtually unknown. The choice of DHEA as the study object is associated with its tendency to form polymorphous structures [15,16]. DHEA is a natural neurohormone, which performs important functions in many processes in the human organism [17]. DHEA has eight different polymorphic forms. Six crystalline forms of DHEA (FI−FVI) were described [18,19], and the forms FVII and FVIII have been described previously [14]. In the present study, we analyze the mechanism of formation of nanoparticles, its connection with the formation of polymorphous nanostructures, the synthesis of the latter as a function of the nature of carrier gas, and the temperature of cryochemical synthesis.

## 2. Results

The main result of this study is the discovery of the dependence of selectivity of produced polymorphous DHEA nanostructures on the nature of carrier gas and conditions of synthesis of nanoparticles involved in the formation of polymorphous structures. Table 1 shows the data on the particle size and on the effect of the nature of carrier gas on the structure and ratio of nanoforms produced under commensurable experimental conditions. As a typical experiment in the dynamic mode, we carried out the optimization of the cryochemical synthesis of the FVII DHEA nanoform. The carrier temperature was 140 °С, the DHEA amount was 200 mg, the condenser temperature was 77К, the normalized gas flow velocity was 0.133 mol/m^2^ s, the carrier gas was CO_2_, the distance from heater to condenser was 13 mm, the mass yield of FVII form was 70%, the average size of nanoparticles was 100 nm. The other carrier gases were studied under conditions commensurate with those used for CO_2_. Besides CO_2_, we used nitrogen and helium as carrier gases.

The yields and sizes of nanoparticles shown in Table 1 were averaged based on the data of several 4-h experiments. The conditions indicated in the table were taken as the basis for carrying out comparable experiments with other carrier gases. The error in determination of the ratio of nanoforms was indicated in the table, average size—10%; overall yield—5%; the above molar flow carrier gas—1.5%.

**Figure 1:**
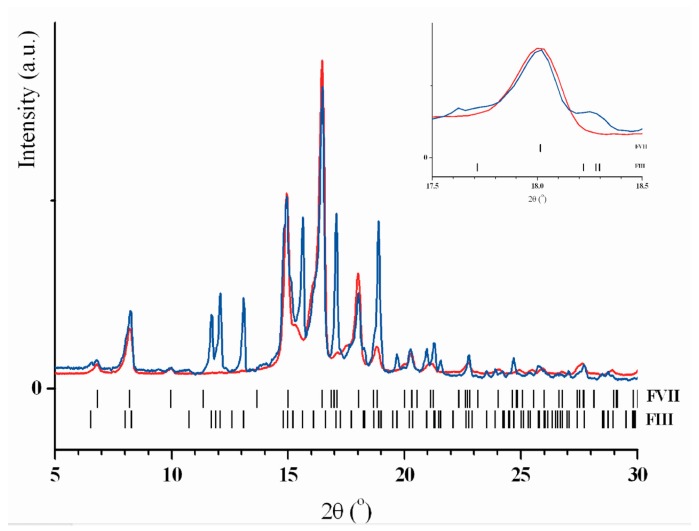
X-ray powder patterns of the cryomodified DHEA.

It is evident from table that the highest yield of the new polymorphous modification FVII was reached when CO_2_ was used as the carrier gas. Under commensurable experimental conditions, the substitution of nitrogen for CO_2_ decreased the content of the structure FVII to 55%, the FIII structure appeared and the FVIII structure remained unchanged. The size of nanoparticles was 120 nm. The FVIII form is yet another DHEA modification we observed earlier, but we failed to determine its crystallographic parameters [14]. In certain experiments with CO_2_, we observed changes in the yield of FVIII with variations in the flow. Considerable changes in the ratio of polymorphous modifications were observed for helium. Figure 1 shows typical X-ray powder patterns of the cryomodified DHEA samples obtained with different gas carriers (CO_2_ and He). The amount of FVIII decreased to 5%, the amount of FVII decreased to 55%. The average particle size was 150 nm.

X-ray powder patterns of the cryomodified DHEA samples obtained with different gas carriers, CO_2_ (red) and He (blue). Vertical black bars show the calculated Bragg peak positions for the polymorphs FVIII and FIII. Inset demonstrates a comparison of standing separately Bragg peak of FVII (hkl = 11–1, 2θ = 18.00°, scaled to one maximum): the wider (red) peak evidences for smaller particle size (see also Table 1).

Substantial changes in the structure of polymorphs were observed in experiments carried out in the static mode in the absence of carrier gas. The size of resulting nanoparticles was 120 nm and the content of the initial FI form decreased by 80% and was transformed to FII. The structures FVII and FVIII were absent. Thus, it is evident that the method of synthesis of nanoparticles in its pure form is insufficient for molecular assembling to produce new polymorphous nanostructures. The third component, carrier gas, and its nature are of great importance.

## 3. Discussion

In the cryochemical strategy of synthesizing nanoparticles of organic drug compounds, which we develop here, the presence of the precursor molecules in the gaseous state under the conditions of high mobility and weak molecular interactions plays the important role. The initial gaseous state allows one to use the approach known in nanotechnology as “from the bottom” [6]. Another factor having the decisive effect on the nanoparticle size is the temperature used for cooling the surface with which the molecules with excessive energy supplied from the heated evaporator interact. Yet another factor associated with the cryochemical synthesis of polymorphs is the form of nano-crystals that ensures the absence of transformations on heating and stability at room temperature. It was found that, when stored at room temperature, the FVII nanostructure retains its form and size for a year [14]. The general feature of the technology we develop is that it combines high and low temperatures in the dynamic mode, which provides the continuous homogeneity in the gas phase and the heterogenic transformations in the condenser. The dynamic mode induces changes that determine the temperature drop, activation of molecular interactions, growth of solid nuclei, and the formation of nanoparticles which under these high-mobility conditions form polymorphous crystalline nano-forms stabilized at low temperatures in the condenser. The main contribution to the size of nanoparticles involved in molecular assembling of nano-forms is made by such factors as the temperature and the presence and the nature of carrier gas, which regulates the energy selection in the system.

In the absence of carrier gas, the mechanism of formation of nanoparticles and nanostructures changes. Being evaporated in vacuum, the molecules experience no intermolecular interactions within their flow because the free path of particles exceeds the distance from the heater to the surface cooled by liquid nitrogen. At their heterogeneous-adsorptive interaction with a cold surface, the particles that possess excessive energy can stabilize, migrate, and be reflected. Under conditions of low temperature and permanent supply of molecules, the reflected particle cannot move freely and is stabilized. Heterogeneous nucleation sites are created. Interaction with original molecules from the evaporator provides the growth of nanoparticles and their assembling to nanostructures. At the transition from low temperatures to room temperature, the most advantageous structures are formed and stabilized in the cryochemical synthesis. The size of crystalline nanoparticles is determined by the stabilization temperature. A certain confirmation to the proposed mechanism of synthesis of nanoparticles and nanostructures is condensation in vacuum on a surface with a temperature higher than 77 К. In the latter case, the particle size increases to reach several micrometers; the yield of FII decreases and the yield of FIII increases.

The dynamic mode of cryochemical synthesis of new polymorphous nanostructures based on combining high and low temperatures and the absence of solvents opens up new possibilities for synthesizing drug substances. The use of drug nanostructures is most promising for the per oral and transdermal introduction. Table 1 shows that the highest selectivity is observed when CO_2_ is used as the carrier gas. Under commensurable conditions, nitrogen and helium decrease the selectivity with respect to FVII, leading to the formation of other structures.

Let us consider these peculiarities based on the concept of competition between activity and selectivity. This principle, i.e., that high activity leads to low selectivity and low activity leads to high selectivity, is used in the analysis of reactions involving free radicals in liquid and gas phases [20].

It can be assumed that, in the case of CO_2_, the high selectivity is formed in the gas phase before contact with the cold surface. The molecules coming out from the hot jet with the inert-gas flow possess excessive energy obtained upon the transition of original crystals to the gaseous state. Such factors as the high mobility of particles in the gas phase, the possibility of the exchange, and loss of energy upon intermolecular interactions and collisions with reactor walls and the temperature drop reduce the excessive energy and subsequent activity.

The additional regulating contribution to the energy exchange is made by the nature of carrier gas. Playing the role of the third particle, the carrier gas, its size, and presence of bonds also favor a decrease in activity. Thus, depending on the nature of carrier gas, the molecules of different activity can interact with the cold surface. The sharp temperature drop disturbs the equilibrium in the stationary flow, and nucleation crystallization sites are formed on the cold surface. Due to the dynamic mode in the nucleation sites, nanoparticles of certain activity are formed and grow further by reacting with original molecules, which provides assembling of the corresponding polymorphous nano-forms. As a result of combination of the above factors, the structures synthesized in the presence of carbon dioxide exhibit the lowest activity and the highest selectivity.

We should note one more difference between linear molecules of СО_2_ and N_2_. During interaction of the flow of DHEA and CO_2_ molecules with the surface of a condenser cooled by liquid nitrogen, some of the CO_2_ molecules precipitate together with DHEA molecules. This may serve as an additional factor that reduces the activity of adsorbed DHEA molecules, which contributes to the formation of nanoparticles under conditions of cryosynthesis and their subsequent transformation into linear structures of the FVII form, which have high selectivity and smaller particle sizes. Other processes take place in the case of N_2_ as an inert carrier gas. In the gas phase, the interaction of DHEA molecules with N_2_ leads to the formation of more active particles. When such active particles interact with a cold surface, the migration of DHEA molecules on the surface remains, which can lead to the formation of two or more nuclei having different activities. Interaction with similar embryos of DHEA molecules from the stream leads to the formation of nanoparticles and their transformation into nanostructures of different compositions and sizes.

The synthesis of nano-crystalline polymorphous modifications for drug substances can be considered as the manifestation the size effect for organic nanoparticles. This effect was also observed for nanoparticles of different metals, being sufficiently well-studied [6].

Substances that are suitable for the strategies used in the work must correspond to certain parameters. Substances should have an oxygen, an amino, and a sulfide group. Substances should not decompose on heating. They should have a melting point of 100–250 °C and form hydrogen bonds. These substances should not have groups that can interact within a gas phase.

Concluding, it should be noted again that, for poorly soluble substances, the therapeutic properties strongly depend on their transformation to polymorphous modifications. The polymorphism changes solubility and dissolution rate, increasing the permeability and bioavailability [4].

## 4. Materials and Methods 

In this study, we used 3β-hydroxy-5-androsten-17-one (DHEA) produced by Acros Organics (Noisy-Le-Grand, France). This substance pertains to group FI with Тm.p. = 150.1 °С and the average particle size of 100 µm. The particle size was determined by optical microscope ERGAVAL (Karl Zeiss, Jena, Germany).

**Figure 2:**
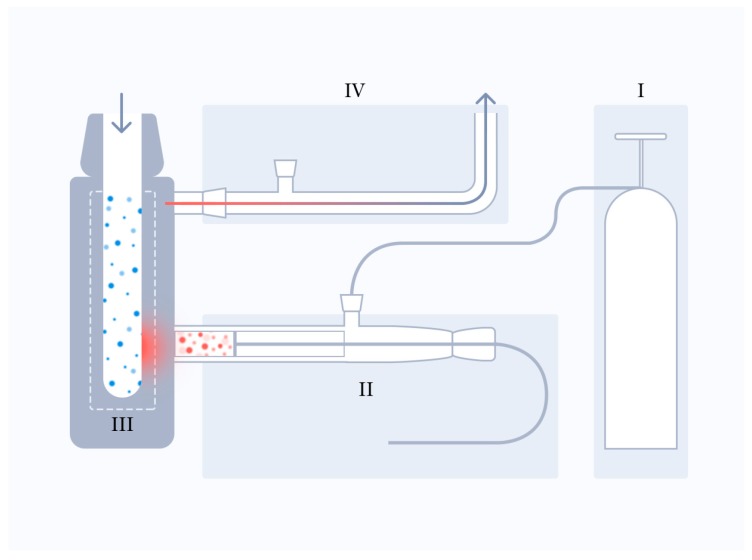
Nanostructure synthesis block-scheme.

Figure 2 shows the synthesis block-scheme corresponding to its dynamic version for the production of nanoparticles and nanostructures. Here, Ι is the block of regeneration of the carrier gas; ΙΙ is the block of sublimation of DHEA molecules, ΙΙΙ is the condenser, i.e., the block of cryostabilization of nanoparticles and nanostructures; ΙV is the vacuum block responsible for the dynamic mode. It was found that cryochemical synthesis of DHEA nanoparticles and nanostructures depends on several parameters. The factors that exert the strongest effect on the structure and size of the resulting particles are the temperature of evaporator and condenser, the velocity and the nature of carrier gas, the heterogeneous and homogeneous mechanisms of nucleation, and growth of structures. The evaporator temperature determines the concentration of the original molecules in the flow. The condenser temperature determines the size of synthesized and stabilized nanoparticles and nanostructures. The velocity and the nature of carrier gas in combination with the nucleation mechanism determine the yield and ratio of the polymorphous nanostructures. The structure of resulting DHEA nanoforms was controlled on a qualitative level by the Fourier transform infrared spectroscope, Tenzor II (Bruker, Rheinstetten, Germany). The quantitative analysis of the structure of nano-forms and their ratio was carried out by the powder X-ray diffraction method (Huber G670, Cu Kα1, λ = 1.54059 Å). The procedures used in this study for determination of the composition and ratio of polymorphous modifications based on XRD method was developed earlier [14].

The average current size of nanoparticles was estimated based on their surface area, all particles were assumed to be spherical. The surface area was determined based on the argon adsorption by the gas-chromatographic method [21].

The nanoparticle size was also confirmed by means of transmission electron microscopy (LEO 912, ABOmega ZEISS, Jena, Germany) and controlled by the atomic force microscopy (Innova Bruker, Rheinstetten, Germany). The particle size of FVII form determined from histogram was 100 ± 10 nm under optimal conditions.

Besides CO_2_, we used nitrogen and helium as the carrier gases. All the gases were dried on molecular sieves Wolfen-Zeosorb 3 A (VEB Laborchemie Apolda, Leipzig, Germany). Experiments in the absence of carrier gas were carried out in high vacuum. Evaporation of the FI form of original DHEA was carried out from a glass ampoule at 140 °С for 4 h. The products were condensed on glass or a polished copper cube cooled with liquid nitrogen. The created degree of vacuum excluded collisions of molecules in the space between the evaporator and the cold surface. The analogous method was often used in studying cryoreactions of metal nanoparticles with different organic compounds [6]. The condensation on a polished surface makes it possible not only to collect reflectance IR spectra at 77 K but also to control the changes occurring at heating.

With the technology we used, the molecules of the original substance were subjected to high and low temperatures and also to changes in the phase state. For DHEA, the earlier investigations by the methods of thin-layer chromatography and mass spectrometry have shown that it retains its original molecular structure during cryochemical synthesis [22].

## 5. Conclusions

The cryochemical strategy of transforming poorly water soluble crystalline drugs, which was applied here to steroid neurohormone DHEA, made it possible to reveal the dependence of polymorphous nanostructures on the nature of inert carrier gas.

The effects of the temperature of the sublimator and condenser, the flow velocity, and the properties of the carrier gas were studied. We discussed peculiarities of cryochemical synthesis of nanoparticles and their transformation to molecular nanostructures. We took into considereation the competition between activity and selectivity, the effect of experimental conditions on the formation of polymorphous nanostructures, and the homogeneous and heterogeneous mechanisms of the syntheses of nanoparticles. We propose considering the synthesis of polymorphous crystalline drug substances as the manifestation of the size effect for organic nanoparticles.

## Figures and Tables

**Table 1:** The effect of the nature of carrier gas on the yield of polymorphous nanostructures.

Carrier Gas	Mass Yield, %	FII %	FIII %	FVII %	FVIII %	Particle Size, nm
СО_2_	75	0	0	70 ± 10	30 ± 10	100
Не	51	0	40 ± 3	55 ± 3	5 ± 3	150
N_2_	54	0	5 ± 3	55 ± 3	40 ± 10	120
Without (vacuum)	58	80 ± 5	20 ± 5	0	0	120

## References

[1] Stegemann S., Leveiller F., Franchi D., Jong H., Linden H. (2007). When poor solubility becomes an issue: From early stage to proof of concept. Eur. J. Pharm. Sci..

[2] Dengale S.J., Grohganz H., Rades T., Löbmann K. (2016). Recent advances in co-amorphous drug formulations. Adv. Drug Deliv. Rev..

[3] Vasconcelos T., Marques S., Neves J., Sarmento B. (2016). Amorphous solid dispersions: Rational selection of a manufacturing process. Adv. Drug Deliv. Rev..

[4] Censi R., Di Martino P. (2015). Polymorph Impact on the Bioavailability and Stability of Poorly Soluble Drugs. Molecules.

[5] Utekhina A.Y., Sergeev G.B. (2011). Organic nanoparticles. Russ. Chem. Rev..

[6] Sergeev G.B., Klabunde K.J. (2013). Nanochemistry.

[7] Ward G.H., Schultz R.K. (1995). Process-induced crystallinity changes in albuterol sulfate and its effect on powder physical stability. Pharm. Res..

[8] Winter G., Boldyreva E., Boldyrev V. (1999). Polymorphs and Solvates of Molecular Solids in the Pharmaceutical Industry. Reactivity of Molecular Solids.

[9] Bruno J.A., Doty B.D., Gustow E., Illig K.J., Rajagopalan N., Sarpotdar P. (1996). Method of Grinding Pharmaceutical Substances. U.S. Patent.

[10] Morozov Y.N., Utekhina A.Y., Shabatin V.P., Chernyshev V.V., Sergeev G.B. (2014). Cryosynthesis of nanosized drug substances. Russ. J. Gen. Chem..

[11] Morozov Y.N., Sergeev G.B. (2016). Method of Producing Nano-Sized Powders of Medicinal Substances and Device Therefor. Patent.

[12] Utehina A.Y., Moscova A.A., Morozov Y.N., Kolotilov P.N., Sergeev B.M., Sergeev G.B. (2011). Cryosynthesis and physico-chemical properties of hormone Δ5 -androstenediol-3β,17β nanoparticles. J. Butlerov Commun..

[13] Sergeev G.B., Sergeev B.M., Morosov Y.N., Chernyshev V.V. (2010). β-Polymorph of phenazepam: A powder study. Acta Crystallogr. Sect. E Struct. Rep. Online.

[14] Chernyshev V.V., Morozov Y.N., Bushmarinov I.S., Makoed A.A., Sergeev G.B. (2016). New Polymorph of Dehydroepiandrosterone Obtained via Cryomodification. Cryst. Growth Des..

[15] Morozov Y.N., Utekhina A.Y., Sergeev G.B., Chernyshev V.V., Chistyakov V.V., Goncharov N.P., Rzheznikov V.M. (2014). A Crystalline Modification of 3β-Hydroxy-5-Androsten-17-FVII, and Method for Production Thereof. Patent.

[16] Labrie F. (2015). All sex steroids are made intracellularly in peripheral tissues by the mechanisms of intracrinology after menopause. J. Steroid Biochem. Mol. Biol..

[17] Kroboth P.D., Salek F.S., Pittenger A.L., Fabian T.K., Frye R.F. (1999). DHEA and DHEA-S: A review. J. Clin. Pharmacol..

[18] Stahly G.P., Bates S., Andres M.C., Cowans B.A. (2006). Discovery of a New Polymorph of Dehydroepiandrosterone (Prasterone) and Solution of Its Crystal Structure from X-ray Powder Diffraction Data. Cryst. Growth Des..

[19] Chang L.C., Caira M.R., Guillory J.K. (1995). Solid state characterization of dehydroepiandrosterone. J. Pharm. Sci..

[20] Semenov N.N., Semenov N.N., Work S. (2005). On Some Problems of Chemical Kinetics and Reaction Ability.

[21] Karnaukhov A.P., Buyanova N.E., Everett D.H., Ottewill R.H. (1969). High productive chromatographic methods for determination of the total and active surface area of catalysts. Surface Area Determination.

[22] Morozov Y.N., Chistyakov D.V., Utekhina A.Y., Astakhova A.A., Goncharov N.P., Sergeeva M.G., Sergeev G.B. (2016). Cryosynthesis and Properties of Dehydroepiandrosterone Hormone Nanoparticles. Pharm. Chem. J..

